# New insights into the important roles of phase seperation in the targeted therapy of lung cancer

**DOI:** 10.1186/s13578-023-01101-8

**Published:** 2023-08-14

**Authors:** Ying Zou, Hongmei Zheng, Yue Ning, Yang Yang, Qiuyuan Wen, Songqing Fan

**Affiliations:** Department of Pathology, The Second Xiangya Hospital, Central South University, Changsha, 410011 Hunan China

**Keywords:** Liquid–liquid phase separation, NSCLC, Targeted therapy, EGFR, YAP

## Abstract

Lung cancer is a complex and heterogeneous disease characterized by abnormal growth and proliferation of lung cells. It is the leading cause of cancer-related deaths worldwide, accounting for approximately 18% of all cancer deaths. In recent years, targeted therapy has emerged as a promising approach to treat lung cancer, which involves the use of drugs that selectively target specific molecules or signaling pathways that are critical for the growth and survival of cancer cells. Liquid–liquid phase separation (LLPS) is a fundamental biological process that occurs when proteins and other biomolecules separate into distinct liquid phases in cells. LLPS is essential for various cellular functions, including the formation of membraneless organelles, the regulation of gene expression, and the response to stress and other stimuli. Recent studies have shown that LLPS plays a crucial role in targeted therapy of lung cancer, including the sequestration of oncogenic proteins and the development of LLPS-based drug delivery systems. Understanding the mechanisms of LLPS in these processes could provide insights into new therapeutic strategies to overcome drug resistance in lung cancer cells.

## Introduction

Lung cancer is the leading cause of cancer-related deaths worldwide, with non-small cell lung cancer (NSCLC) accounting for approximately 85% of all lung cancer cases [1]. The primary treatment options for NSCLC include surgery, chemotherapy, and radiotherapy. However, in recent years, targeted therapy and immunotherapy have emerged as promising new approaches to treating lung cancer [2–4]. Targeted therapies, such as EGFR inhibitors and ALK inhibitors, selectively target cancer cells while sparing normal cells, resulting in improved efficacy and fewer side effects compared to traditional chemotherapy. Immunotherapy, such as immune checkpoint inhibitors, works by stimulating the body’s immune system to recognize and attack cancer cells [5–7]. While these therapies have shown significant promise in the treatment of lung cancer, more research is needed to optimize their use and identify new therapeutic targets. Liquid–liquid phase separation (LLPS) is essential for various cellular functions, including the formation of membraneless organelles, the regulation of gene expression, and the response to stress and other stimuli [8]. Recent studies have shown that LLPS plays a crucial role in targeted therapy of lung cancer, including the sequestration of oncogenic proteins and the development of LLPS-based drug delivery systems [3, 9–11].

## LLPS and its role in cellular function

LLPS occurs through the assembly of multivalent interactions between disordered regions of proteins or RNA molecules, leading to the formation of dense liquid droplets. There is emerging evidence suggesting that the formation of liquid droplets through LLPS plays a critical role in the response of lung cancer cells to targeted therapies [3, 7, 12–18]. For example, it has been shown that in NSCLC cells with EGFR mutations, the formation of liquid droplets containing mutant EGFR leads to the sequestration of TKIs, reducing their effectiveness [19, 20]. Understanding the mechanisms of LLPS in these processes could provide insights into new therapeutic strategies to overcome drug resistance in lung cancer cells. In addition, studies have shown that LLPS is also involved in the splicing process of mRNA, leading to the development of tumors [21]. Moreover, LLPS has also been shown to play a role in the formation and maintenance of drug-resistant cancer stem cells, which are thought to contribute to tumor recurrence and metastasis [22, 23]. Targeting LLPS in these cells may provide a new therapeutic strategy for improving the effectiveness of targeted therapies in lung cancer. Overall, the emerging role of LLPS in the response of lung cancer cells to targeted therapies presents an exciting avenue for further research into the mechanisms of cancer and the development of novel therapeutic strategies [11].

## Mechanisms of LLPS in lung cancer

The aberrant regulation of LLPS has been increasingly recognized as a critical factor in the development and progression of lung cancer [15]. The major examples of advances in LLPS-based resistance against lung cancer are summarized in Table 1.

**Table 1:** Main mechanisms of LLPS in lung cancer

Types	Representative substance	Mechanisms	References
Tumor suppressor protein	P53, PTEN	Mutations in them can disrupt the formation of their liquid droplets, leading to impaired DNA repair and increased genomic instability	[20, 24]
NONO		Function as sites of DNA repair and signal transduction,influence the positioning of EGFR NONO promotes TAZ LLPS	[19]
DUB	USP42	Govern the phase separation of PLRG1 to promote tumorigenesis	[21]
Non-membranous compartment	Stress granule	Improve survival advantages and chemotherapy resistance of malignant tumor cells	[33]
Phosphatase	SHP2	SHP2mut induces strong LLPS to recruit SHP2WT and promote ERK activation	[26]
Lysine methyltransferase	EZH2	Myristoylation-mediated phase separation of EZH2 activates STAT3 signaling and promotes lung tumor growth	[27]
	YAP, TAZ	Undergo LLPS and accumulate in the nucleus to drive oncogenic genes	[28]
RTK	ALK	LLPS of EML4-ALK actives several signalings in lung cancer	[29]

In lung cancer cells, LLPS can occur through the interaction of disordered regions of proteins, such as the non-POU domain-containing octamer-binding protein (NONO), with RNA molecules. The resulting liquid droplets can function as sites of biochemical reactions and contribute to various cellular processes, including DNA repair and signal transduction [20]. One key mechanism by which LLPS contributes to lung cancer development is through the formation of liquid droplets that sequester tumor suppressor proteins. For example, the tumor suppressor protein p53 has been shown to undergo LLPS in response to DNA damage, forming liquid droplets that recruit and activate downstream effectors of the DNA damage response [20]. However, in lung cancer cells, mutations in p53 can disrupt the formation of these droplets, leading to impaired DNA repair and increased genomic instability. Similarly, other suppressor proteins such PTEN have also been shown to undergo LLPS, and mutations in these genes can impair their ability to form liquid droplets and contribute to cancer development [24].

In addition of tumor suppressor proteins, LLPS of NONO has been found to recruit and enhance the interaction between EGFR and DNA-dependent protein kinase (DNA-PK), a key protein in the non-homologous end joining (NHEJ) pathway of DNA double-strand break repair. This interaction leads to the activation of pT2609-DNA-PK and promotes NHEJ-mediated DNA repair, which contributes to tumor radioresistance [19, 30–32].

Furthermore, the phenomenon of liquid–liquid phase separation (LLPS) emerges as a key player in the intricate landscape of anti-tumor immune responses in lung cancer. Recent studies have shed light on the disruptive effects of perturbing the phase separation of KAT8–IRF1, resulting in a reduction in PD-L1 expression and a concomitant enhancement of the anti-tumor immune response [23]. Remarkably, LLPS also exerts a significant influence on mRNA splicing, a process intricately linked to tumorigenesis. Notably, the deubiquitylase USB42 has been identified as a critical regulator of phase separation within the spliceosome component PLRG1. This regulatory mechanism has been demonstrated by Liu et al. to govern a multitude of mRNA splicing events that encompass diverse cellular functions. The clinical implications of these findings are profound, as they shed light on the potential prognostic value of LLPS-related mRNA splicing events in lung cancer patients [21].

In addition, a number of recent studies have shown that LLPS is involved in the signaling activation of lung cancer carcinogenic pathways such as EGFR, ALK and KRAS [19, 27, 29, 34]. The following is the detailed process of LLPS participating in the activation of carcinogenic signals in lung cancer.

## LLPS in EGFR pathway

The activation of the EGFR signaling pathway through LLPS-mediated mechanisms is linked to the dysregulation of several downstream oncogenic pathways. In NSCLC, the activation of EGFR signaling can occur through several mechanisms, including mutations in the EGFR gene, gene amplification, or overexpression [19]. For example, the accumulation of EGFR in the nucleus can lead to the overexpression of several transcription factors, including c-Myc [25], which is associated with tumor growth and angiogenesis. The LLPS-mediated recruitment of EGFR to the transcriptional co-activator CBP/p300 complex has also been shown to promote the transcription of EGFR target genes involved in cell cycle progression, such as cyclin D1 and B-Myb [25, 35, 36]. Additionally, the activation of EGFR signaling can lead to the upregulation of COX-2, an enzyme involved in the production of prostaglandins that promote tumor growth, invasion, and angiogenesis [35]. LLPS of NONO has been shown to enhance the recruitment of EGFR to the COX-2 promoter, resulting in increased COX-2 expression and promoting tumor progression [36].

**Fig. 1:**
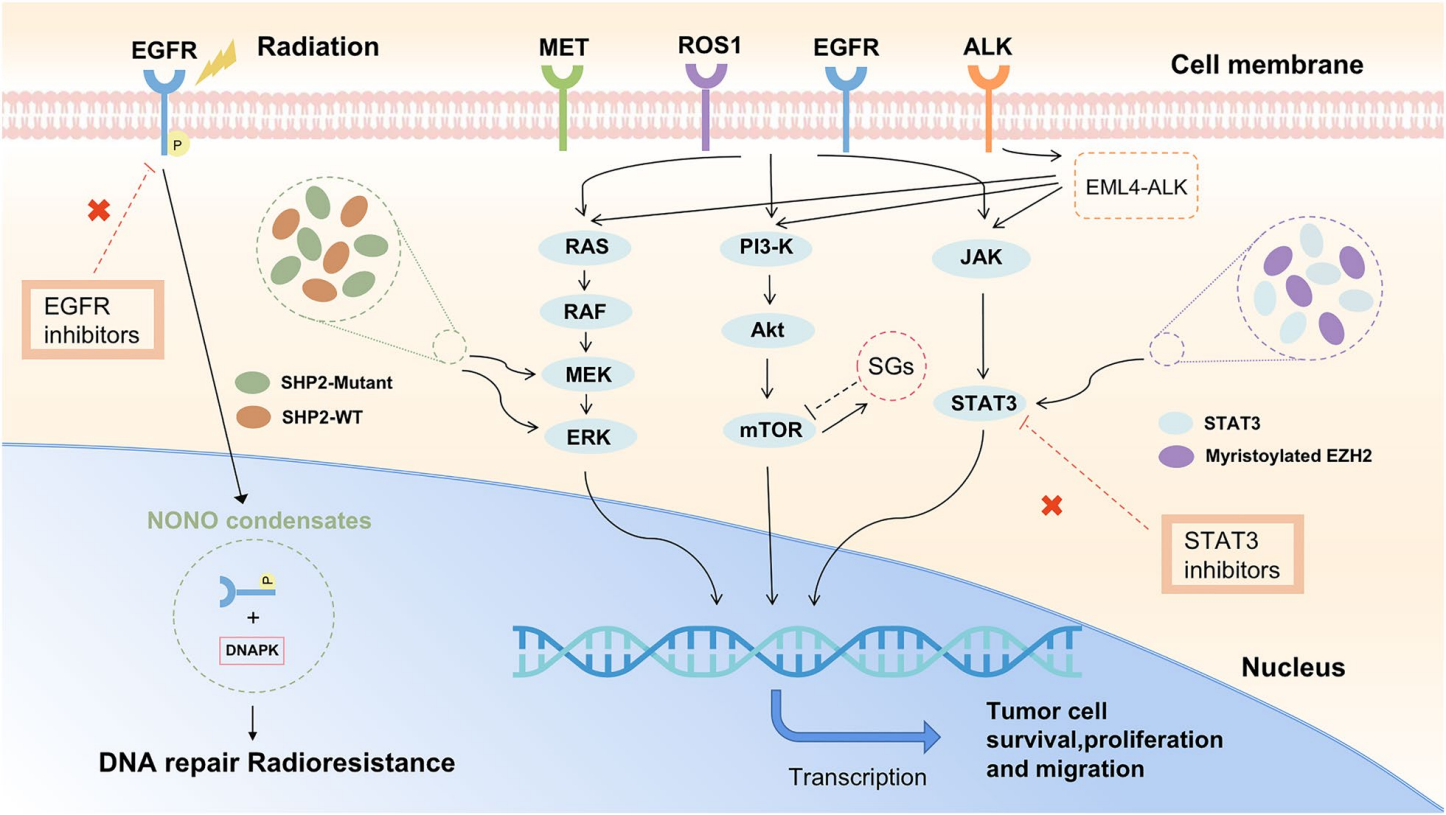
NONO condensates recruit DNA-PK and nEGFR, promote NHEJ-mediated DNA repair and induce resistance to radiation in tumors.SHP2mut induces strong LLPS to recruit SHP2WT and promote ERK activation. The formation of SGs interacts with mTOR. LLPS of EML4-ALK actives several signalings in lung cancer. Myristoylation-mediated phase separation of EZH2 activates STAT3 signaling and promotes lung tumor growth

Targeting the aberrant regulation of LLPS in lung cancer cells could provide new opportunities for the development of more effective therapies for this devastating disease. The formation of LLPS droplets can recruit and activate EGFR and downstream signaling proteins, leading to increased cell proliferation, survival, angiogenesis, invasion, and radioresistance [37, 38]. These are summarized in Fig. 1. LLPS of NONO can also enhance the interaction between EGFR and DNA-PK, promoting NHEJ-mediated DNA repair [19, 30–32]. Dysregulation of the EGFR signaling pathway through LLPS-mediated mechanisms can lead to the upregulation of transcription factors, such as c-Myc, and COX-2, promoting tumor growth, invasion, and angiogenesis [25, 39].

Overall, LLPS is an emerging area of research that provides new insights into the regulation of intracellular signaling pathways, and understanding the role of LLPS in cancer may lead to the development of new therapeutic approaches for the treatment of lung cancer.

## LLPS in ALK and KRAS pathway

KRAS mutations are found in approximately 35% of all lung adenocarcinomas, making it a paramount driver of this particular lung cancer subtype [40]. EML4-ALK, an oncogenic fusion protein resulting from chromosomal translocations, has been extensively studied in the context of lung adenocarcinoma [41].

Recent discoveries have shed light on a captivating phenomenon: the liquid–liquid phase separation (LLPS) of EML4-ALK, which not only contributes to tumorigenesis but also exerts a profound influence on the KRAS signaling pathway [29]. The fusion of the echinoderm microtubule-associated protein-like 4 (EML4) and anaplastic lymphoma kinase (ALK) generates an oncogenic fusion protein with transformative potential [29]. However, recent investigations have unveiled an additional layer of complexity: the propensity of EML4-ALK to undergo LLPS, creating distinct subcellular compartments that harbor a unique signaling microenvironment. EML4-ALK condensates can modulate the KRAS signaling pathway, amplifying the oncogenic potential of this cascade. Through direct physical interactions and the recruitment of critical signaling effectors, EML4-ALK condensates facilitate the activation of downstream effectors in the KRAS pathway, ultimately leading to dysregulated cellular proliferation and survival. Additionally, the spatial organization and confinement of KRAS effectors within EML4-ALK condensates potentiate their signaling output, amplifying the oncogenic signals propagated by KRAS [29, 34].

Moreover, LLPS of SHP2 is involved in the regulation of the MAPK signaling pathway, which is frequently dysregulated in cancer, including lung cancer. Dysregulation of SHP2 contribute to the development and progression of lung cancer.

SHP2 mutants undergo LLPS in the cytoplasm of cancer cells, enhancing MEK1/2 and ERK1/2 phosphorylation levels, and activating MAPK pathway, leading to increased proliferation, survival, and metastasis of lung cancer cells. Disrupting the LLPS of SHP2 and MAPK may be an effective strategy for inhibiting the oncogenic signaling pathways that promote the development and progression of lung cancer [26, 32, 43–45].

## LLPS in the JAK-STAT3 signaling pathway

EZH2, a critical component of the Polycomb Repressive Complex 2 (PRC2), is known for its role in epigenetic regulation. It catalyzes the trimethylation of histone H3 at lysine 27 (H3K27me3), leading to gene silencing [46–48]. On the other hand, STAT3 is a transcription factor involved in numerous cellular processes, including cell proliferation, survival, and immune responses [49]. Dysregulation of both EZH2 and STAT3 has been implicated in the development and progression of lung cancer [47, 50].

Emerging evidence has revealed that the myristoylation modification of EZH2 enables its phase separation, a process by which intracellular components segregate into liquid-like condensates [27]. Myristoylation, the addition of a myristoyl group, facilitates the self-assembly of EZH2 into condensates, forming specialized compartments within the cell. Remarkably, the phase separation of myristoylated EZH2 has been found to compartmentalize STAT3 within the condensates. This sequestration of STAT3 in the EZH2 condensates leads to the sustained activation and enhanced transcriptional activity of STAT3 [27].

Targeting the myristoylation process, disrupting EZH2-STAT3 interactions within the condensates, or modulating the properties of EZH2 condensates may hold promise for developing novel therapeutic interventions.

## LLPS in PI3-K-AKt-mTOR signaling pathway

One of the concepts that formed stress granules was the LLPS process [51]. Stress granules are dynamic and membraneless organelles that form in response to cellular stress, such as oxidative stress, nutrient deprivation, or viral infection. These granules act as hubs for mRNA and protein sequestration, enabling their storage and protection during stress conditions. The dysregulation of stress granule dynamics has been implicated in numerous diseases, including cancer [52–54].

Stress granules dynamically interact with a key component of lung cancer’s pathway, mTOR and its regulators, influencing its localization, activity, and downstream signaling. This interplay modulates cellular responses to stress and contributes to the adaptive and survival mechanisms of lung cancer cells [33].

## LLPS in Hippo signaling pathway

**Fig. 2:**
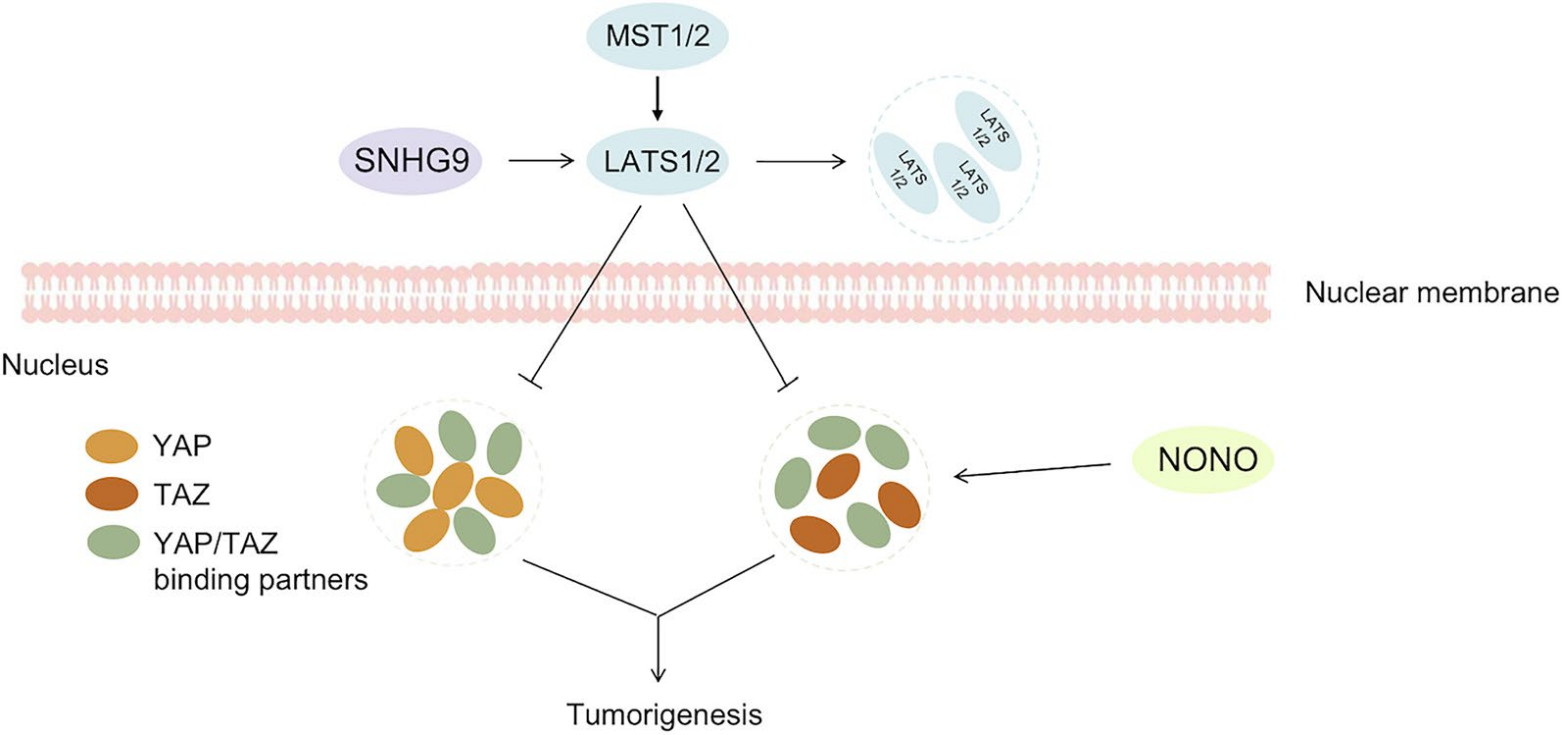
YAP/TAZ undergo LLPS, forming liquid condensates with their relative co-activators, enhanced transcriptional activity, leading to tumorigenesis.SNHG9 drives the liquid droplet formation of LATS1 and inhibits the Hippo pathway. NONO promotes TAZ LLPS

The dysregulation of the Hippo signaling pathway, specifically the activation of downstream effectors Yes-associated protein (YAP) and transcriptional co-activator with PDZ-binding motif (TAZ), has been implicated in the development and progression of NSCLC [28, 55]. (These are summarized in Fig. 2). YAP and TAZ are normally inhibited by the upstream kinase cascade, which includes MST1/2 and LATS1/2, resulting in their cytoplasmic retention and degradation by the ubiquitin-proteasome system [56, 57]. However, in NSCLC, this regulatory mechanism is often disrupted, leading to the accumulation of YAP and TAZ in the nucleus where they can interact with a range of transcription factors to drive gene expression programs involved in cell proliferation, survival, and invasion [28]. YAP and TAZ have been shown to regulate the expression of various oncogenic genes involved in cell growth and metastasis and promote resistance to chemotherapy and targeted therapy in NSCLC [28]. Targeting YAP and TAZ has emerged as a promising therapeutic strategy for NSCLC. Recent evidence suggests that TAZ exerts its transcriptional regulatory functions through the formation of nuclear condensates via LLPS. This process allows for the compartmentalization of TAZ-binding partners to enhance transcriptional activity [39]. TAZ has been shown to form phase-separated droplets in vitro and liquid-like nuclear condensates in vivo, and this ability is negatively regulated by Hippo signaling via LATS-mediated phosphorylation [58]. On the other hand, YAP exhibits differences from TAZ in terms of its ability to undergo LLPS, and it requires specific crowding agents to form droplets. The nuclear factor NONO has also been shown to promote TAZ LLPS and activation in driving the oncogenic transcriptional program [59].

Danfeng Cai and colleagues have demonstrated that YAP is also involved in the LLPS process, which leads to the formation of liquid-like condensates in the nucleus within seconds of hyperosmotic stress. These condensates compartmentalized the YAP transcription factor TEAD1 and other YAP-related co-activators, such as TAZ, leading to the transcription of YAP-specific proliferation genes. Additionally, a tumor-promoting long non-coding RNA (lncRNA) known as small nucleolar RNA host gene 9 (SNHG9) has been found to identified to drive the liquid droplet formation of Large Tumor Suppressor Kinase 1 (LATS1) and inhibits the Hippo pathway. When the Hippo pathway is activated, upstream regulators such as MST, MAP4K, and TAOK phosphorylate and activate LATS1, which in turn sequesters YAP in the cytoplasm by facilitating YAP phosphorylation (S127) and leading to its degradation. During cancer development, lipid signaling related to PA is activated, resulting in the upregulation of SNHG9. These factors promote LATS1 LLPS, decrease LATS1 phosphorylation (S909), and reduce kinase activity in the cytoplasm, leading to YAP activation [60]. However, the development of effective YAP/TAZ-targeted therapies for NSCLC is still in its early stages, and further research is needed to fully understand the role of YAP and TAZ in NSCLC and identify the best approaches to targeting them.

Overall, the dysregulation of the Hippo signaling pathway and the LLPS of YAP and TAZ have important implications for the development of new therapeutic approaches for NSCLC. Disrupting the YAP/TAZ signaling axis and their LLPS may be an effective strategy for inhibiting the oncogenic signaling pathways that promote the development and progression of NSCLC.

## Potential targets for LLPS-based therapies in lung cancer

The role of LLPS in lung cancer progression has shed light on several potential targets for the development of LLPS-based therapies. One promising target is the EGFR signaling pathway, which is frequently dysregulated in lung cancer and plays a critical role in cancer cell proliferation, migration, and survival. Targeting the LLPS-mediated activation of EGFR and its downstream signaling pathways, such as MAPK, has emerged as a promising strategy for inhibiting tumor growth and metastasis. LLPS of other oncoproteins, such as SHP2, has been shown to play a role in lung cancer progression and resistance to therapy, making it another potential target for LLPS-based therapies. In addition to targeting specific oncoproteins, modulation of the biophysical properties of the cancer cell microenvironment through LLPS inhibitors could represent a novel approach for limiting tumor growth and metastasis. For instance, targeting the formation of liquid droplets within the tumor microenvironment, such as stress granules or P-bodies, could inhibit cancer cell survival and migration [61, 62]. LLPS-based therapies could also sensitize cancer cells to conventional therapies, such as chemotherapy and radiation, by enhancing DNA damage repair and inhibiting resistance mechanisms [19, 26, 30–32, 37, 38].

Targeted therapies that focus on inhibiting the LLPS-mediated activation of the EGFR pathway represent a promising approach for treating lung cancer. One potential strategy is the use of LLPS inhibitors that can disrupt the formation of EGFR signaling complexes and inhibit downstream signaling pathways. Another approach is to target specific proteins involved in the LLPS-mediated activation of the EGFR pathway, such as the NOPOU, which plays a critical role in the nuclear accumulation and activation of EGFR in response to radiation. Small molecule inhibitors of NONO and other LLPS-associated proteins could be a potent therapeutic strategy for inhibiting the activation of the EGFR pathway and sensitizing cancer cells to conventional therapies [30, 31–32].

LLPS-based targeted therapies of SHP2 and other oncoproteins in lung cancer represent another promising therapeutic approach. SHP2 is an oncoprotein that plays a critical role in the activation of the MAPK pathway and the promotion of cell proliferation and survival. Small molecule inhibitors of SHP2 have been developed and shown to be effective in preclinical studies, providing a potential avenue for the development of LLPS-based therapies targeting SHP2 in lung cancer. Other oncoproteins involved in the LLPS-mediated activation of oncogenic pathways, such as MET and ROS-1, are also promising targets for LLPS-based therapies. As research into the mechanisms and functions of LLPS in lung cancer continues, the development of targeted therapies that specifically disrupt the formation and activity of oncoprotein droplets could have significant therapeutic implications for patients with this devastating disease [26, 43]. The use of LLPS-based therapies in combination with conventional therapies, such as chemotherapy and radiation, could also enhance the efficacy of current treatments and reduce the incidence of resistance mechanisms.

In addition to targeting specific oncoproteins and their interactions with the ECM, LLPS-based therapies could also be used to sensitize lung cancer cells to conventional therapies such as chemotherapy and radiation. One potential strategy is to use LLPS inhibitors to enhance the efficacy of DNA damage repair, which is critical for cancer cell survival and resistance to therapy. By inhibiting LLPS-mediated repair mechanisms, such as the formation of DNA repair foci, LLPS inhibitors could sensitize cancer cells to DNA damage induced by conventional therapies, leading to improved treatment outcomes [26].

In summary, LLPS has emerged as an important regulator of cellular processes in lung cancer, and its dysregulation plays a critical role in tumor growth, metastasis, and resistance to therapy. Targeting the LLPS-mediated activation of oncoproteins and their interactions with the cancer cell microenvironment represents a promising approach for developing novel therapies to improve the treatment of lung cancer. As research in this area continues, the development of targeted LLPS inhibitors and their use in combination with conventional therapies holds great promise for improving treatment outcomes and reducing the morbidity and mortality associated with this devastating disease.

## Advances in LLPS-based resistance against lung cancer

Recent advances in LLPS-based resistance against lung cancer have shown the potential of this approach in overcoming drug resistance and improving patient outcomes [7, 53, 63–65]. The key advantage of LLPS-based resistance is the ability to selectively target cancer cells while sparing healthy cells. This approach has been shown to be effective against various types of lung cancer, including NSCLC and small cell lung cancer (SCLC). LLPS-based resistance involves the manipulation of protein-protein interactions that regulate the formation of liquid droplets within cells. By targeting these interactions, it is possible to disrupt the formation of liquid droplets that contribute to the survival of cancer cells [25, 57, 63, 53, 64, 66]. The major examples of advances in LLPS-based resistance against lung cancer are summarized in Table 2.

**Table 2:** Examples of advances in LLPS-based resistance against lung cancer

Targets	Types	Functions	References
Inhibitors related to the YAP phase seperation process	EVG	Disrupt the LLPS of the SRC-1 protein	[66]
	Verteporfin	Sequester YAP in the cytoplasm and disrupt the formation of YAP-containing liquid droplets in cancer cells	[63]
	Peptidomimetics	Disrupt the interaction between YAP and the transcriptional co-activator TEAD	[53]
	YAP-Tead interaction inhibitors	Disrupt the interaction between YAP and the transcriptional co-activator TEAD	[53]
Inhibitors related to the EGFR phase seperation process	SHP099	SHP2 inhibitor	[57]
Drugs affecting the process of LLPS	1,6-Hexanediol	Disrupt the LLPS process of various proteins	[25]
Delivery systems	Liposomes	Encapsulate a variety of drugs and other therapeutic agents	[64]

A promising target is the YAP protein, which is involved in the formation of liquid droplets that promote the survival of cancer cells. The efficacy of EVG in the inhibition of cancer cell growth through the targeting of SRC-1/YAP/TEAD droplets in a YAP-dependent manner has been demonstrated by selective disruption of the liquid–liquid phase separation (LLPS) of SRC-1 [66]. And there are several scientific studies that support the view that various compounds can inhibit YAP droplets in cancer cells.

Targeting the oncogenic signaling pathway has also been the primary focus of LLPS-based therapies, and several inhibitors have been developed to block EGFR accumulation in the nucleus and disrupt its oncogenic functions. For instance, the liquid-like condensates formed by NONO can be disrupted by 1,6-Hexanediol, which is known to disrupt hydrophobic interactions in such droplets [30]. Moreover, the SHP2 inhibitor, SHP099, has been shown to effectively suppress the growth of KRAS-mutant NSCLC tumors in vitro and in vivo models [67]. Additionally, LLPS inhibitors have also shown promise in modulating the tumor microenvironment, leading to improved immunotherapeutic responses and treatment outcomes [68, 69].

Targeted drug delivery systems also provide a promising approach for treating lung cancer, taking advantage of the unique properties of LLPS. For example, liposomes can be designed to recognize specific cell surface markers or to respond to changes in the local environment of tumor tissues, which can enhance their accumulation and internalization in lung cancer cells [64].

Despite the early stages of development, the potential for personalized cancer treatment through LLPS-based targeted therapies is significant, but challenges remain, such as identifying novel targets and optimizing LLPS inhibitors for clinical use. However, with further research, LLPS-based targeted therapies could become a cornerstone in the treatment of lung cancer, offering improved outcomes and reduced toxicities.

## Challenges and future directions for LLPS-based therapies in lung cancer

LLPS-based therapies have shown great promise in lung cancer treatment. However, several challenges that must be addressed to translate these therapies into clinical practice. A major challenge is the lack of understanding of the specific mechanisms of LLPS in lung cancer and the complexity of signaling pathways involved. The development of efficient and specific LLPS inhibitors that can selectively target cancer cells is another challenge [7, 65]. Resistance to LLPS-based therapies is also a concern due to the heterogeneity of lung cancer and the adaptability of cancer cells [14]. Safety and toxicity of LLPS inhibitors need to be fully evaluated in humans, as many of these inhibitors have not yet undergone clinical trials [70].

To overcome these challenges, future research efforts should focus on further elucidating the mechanisms of LLPS in lung cancer and identifying novel LLPS regulators and targets. Advanced imaging techniques and organoid cultures could be used to achieve this goal. Comprehensive studies to evaluate the safety and efficacy of LLPS inhibitors in clinical settings are also needed. Biomarkers can be used to identify patient populations that are most likely to respond to LLPS-based therapies, and combination therapies that can overcome resistance mechanisms [71, 72].

Finally, personalized LLPS-based therapies based on the unique characteristics of individual tumors can potentially improve treatment outcomes and minimize adverse effects. In summary, despite the challenges, LLPS-based therapies hold great potential for improving lung cancer treatment, and continued research in this area is crucial for the development of effective and safe targeted therapies. Combining LLPS-based therapies with immunotherapies is another potential direction for future research [71, 73]. LLPS inhibitors can modulate the tumor microenvironment and enhance the efficacy of immunotherapies by improving immune cell infiltration. Finally, personalized LLPS-based therapies based on the unique characteristics of individual tumors can potentially improve treatment outcomes and minimize adverse effects.

In summary, while LLPS-based therapies hold great potential for the treatment of lung cancer, several challenges need to be overcome before they can be successfully translated into clinical practice. These include the need for a deeper understanding of LLPS mechanisms and signaling pathways in lung cancer, the development of more efficient and specific LLPS inhibitors, and the evaluation of the safety and toxicity of these inhibitors. Nevertheless, ongoing research efforts, such as the development of new technologies and experimental models, as well as the exploration of combination therapies, offer promising avenues for overcoming these challenges. With further advancements and innovations in LLPS-based therapies, there is hope for significant improvements in the clinical outcomes of lung cancer patients.

## Discussion and future perspectives

The dysregulation of LLPS in lung cancer provides an attractive target for the development of novel therapies. However, several challenges must be overcome to successfully translate LLPS-based therapies into clinical practice. The development of specific LLPS inhibitors and effective drug delivery systems, as well as the identification of robust biomarkers, are crucial for the development of successful LLPS-based therapies. Despite these challenges, the future prospects of LLPS-based therapies in the treatment of lung cancer are promising. Advances in our understanding of the biophysical properties of cancer cells and the underlying mechanisms of LLPS in cancer have created new opportunities for the development of targeted therapeutic strategies. LLPS-based therapies targeting specific oncoproteins and oncogenic signaling pathways may improve the efficacy and reduce the toxicity of current treatment strategies. Additionally, the integration of LLPS-based therapies with other modalities, such as immunotherapy and chemotherapy, may provide a synergistic effect and further improve clinical outcomes. Furthermore, the development of LLPS-based therapies may have implications beyond lung cancer. Dysregulation of LLPS is a common feature of many types of cancer, suggesting that the strategies developed for lung cancer may be applicable to other cancer types as well. The development of LLPS-based therapies may provide a new avenue for the treatment of cancers that are resistant to current treatment strategies.

In conclusion, targeting LLPS represents a promising avenue for the development of novel therapies in the context of lung cancer. Significant a﻿dv﻿a﻿nc﻿eme﻿nt﻿s in our understanding of the biophysical properties of the cancer cell microenvironment and the mechanisms of LLPS in cancer have created opportunities for the development of innovative therapeutic strategies. Despite existing challenges, the future prospects of LLPS-based therapies in the targeted treatment of lung cancer are encouraging and offer substantial potential for enhancing clinical outcomes and ultimately mitigating the burden of this debilitating disease.

## Data Availability

Not applicable.

## Abbreviation

LLPS: Liquid–liquid phase separation
